# Conserved developmental expression of *Fezf *in chordates and *Drosophila *and the origin of the *Zona Limitans Intrathalamica *(ZLI) brain organizer

**DOI:** 10.1186/2041-9139-1-7

**Published:** 2010-09-01

**Authors:** Manuel Irimia, Cristina Piñeiro, Ignacio Maeso, José Luis Gómez-Skarmeta, Fernando Casares, Jordi Garcia-Fernàndez

**Affiliations:** 1 Departament de Genètica and Institut de Biomedicina (IBUB), Universitat de Barcelona, Barcelona, Spain; 2 Centro Andaluz de Biología del Desarrollo (CABD), CSIC-Universidad Pablo de Olavide. Campus UPO, Ctra. de Utrera km1, E-41013 Sevilla, Spain

## Abstract

**Background:**

The *zona limitans intrathalamica *(ZLI) and the isthmus organizer (IsO) are two major secondary organizers of vertebrate brain development. These organizers are located at the interface of the expression domains of key patterning genes (*Fezf*-*Irx *and *Otx*-*Gbx*, respectively). To gain insights into the evolutionary origin of the ZLI, we studied *Fezf *in bilaterians.

**Results:**

In this paper, we identified a conserved sequence motif (Fezf box) in all bilaterians. We report the expression pattern of *Fezf *in amphioxus and *Drosophila *and compare it with those of *Gbx*, *Otx *and *Irx*. We found that the relative expression patterns of these genes in vertebrates are fully conserved in amphioxus and flies, indicating that the genetic subdivisions defining the location of both secondary organizers in early vertebrate brain development were probably present in the last common ancestor of extant bilaterians. However, in contrast to vertebrates, we found that *Irx*-defective flies do not show an affected *Fezf *expression pattern.

**Conclusions:**

The absence of expression of the corresponding morphogens from cells at these conserved genetic boundaries in invertebrates suggests that the organizing properties might have evolved specifically in the vertebrate lineage by the recruitment of key morphogens to these conserved genetic locations.

## Background

**Figure 1:**
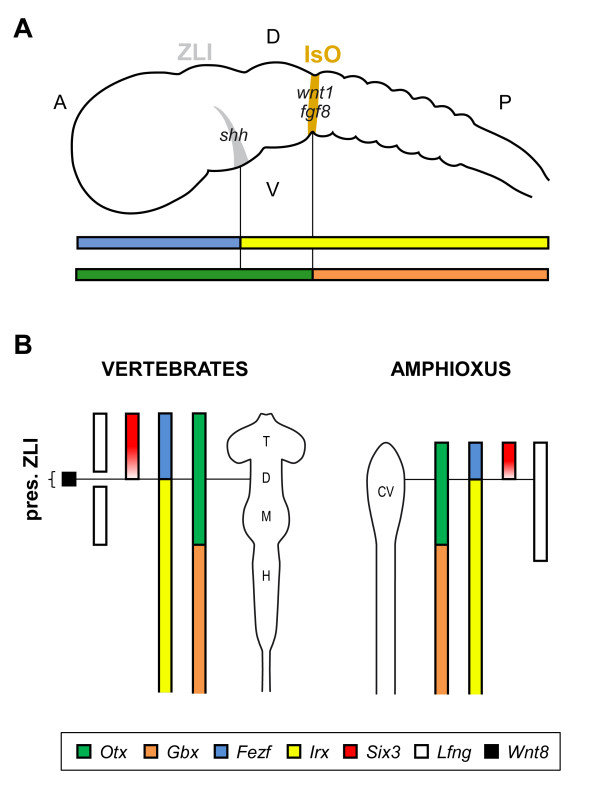
**Schematic view of a generalized vertebrate CNS**. **(A) **Topological location of the ZLI and IsO organizers and the key genes determining their position in a schematic view of a generalized developing vertebrate central nervous system. The most anterior of the internal brain secondary organizers, the ZLI, develops within the diencephalon, at the interface of the expression of *Fezf *(blue) and *Irx *(yellow). The IsO organizer will be located at the midbrain hindbrain boundary (MHB), delimited by the expression of members of the *Otx *(green) and *Gbx *(orange) gene families. **(B) **Scheme of comparative gene expression domains in schematic representations of (left) prototypical vertebrate and (right) amphioxus larva brains (based on [58]). Whereas some patterning genes involved in ZLI location have a conserved relative expression pattern, others like *Lfng*/*Fng *(white) or *Wnt8 *(black) do not. CV = cerebral vesicle; D = diencephalon; H = hindbrain; M = midbrain; T = telencephalon. Note that adult/larval brain structures from vertebrates and amphioxus are not fully comparable.

Secondary morphogenetic organizers are located at the boundaries of major vertebrate brain compartments, and play essential roles in the development of the highly complex vertebrate brain. The two main brain internal organizers are the isthmus organizer (IsO) and the *zona limitans intrathalamica *(ZLI). The IsO is located in the midbrain-hindbrain boundary (MHB), at the abutting expression domains of *Otx *and *Gbx*, and the ZLI develops within the diencephalon, between the prethalamus and thalamus, at the boundary of *Fezf *and *Irx *gene expression domains (Figure 1A). As is typical for organizers, cells from these structures are the source of diffusible signaling factors that determine the further development of the adjacent cellular compartments. ZLI cells characteristically secrete *Shh *[1,2], whereas the IsO typically releases *Fgf8 *and *Wnt1 *[3].

*Bona fide *IsO and ZLI organizers are present in all vertebrates, including basal living agnathans [4,5]; however, the absence of the key morphogens at analogous topological positions [6-9] suggests that comparable signaling centers are not present in invertebrates with a central nervous system (CNS), including amphioxus, a basal chordate considered to be the best living proxy to the vertebrate-invertebrate ancestor [5]. Like vertebrates, amphioxus has a dorsal hollow neural tube that forms from a neural plate. However, the amphioxus brain is relatively simple, consisting only of a putative non-subdivided diencephalon, a *Hox*-patterned hindbrain and perhaps a small midbrain [10].

Despite the lack of internal brain organizers in invertebrates, previous observations suggested that the interface between the abutting expression domains of the *Otx/otd *and *Gbx/unpg *genes, which determines the positioning of the MHB (Figure 1), is ancestral to all bilaterians [11,12]. By contrast, much less is known about the evolutionary origin of the other major anterior-posterior brain subdivision and the ZLI, and how the two organizers physically related to each other originally. Recently, the zinc-finger *Fezf *gene family was reported to have a primary role in establishing the ZLI in vertebrates [13-15]. *Fezf *is expressed exclusively in the most anterior part of the brain, and its caudal expression abuts that of *Irx *genes. The interface of the *Fezf *and *Irx *expression domains delineates the border between the prethalamus and thalamus at the vertebrate diencephalon, and the position at which the ZLI will develop (Figure 1A). However, no studies have yet investigated the origin of the ZLI in organisms other than vertebrates. To gain insights into these questions, we characterized the *Fezf *gene in the basal chordate amphioxus and in the protostome *Drosophila melanogaster*. We analyzed their developmental expression patterns and compared them with those of the *Irx*, *Otx *and *Gbx *genes. Strikingly, we found that the relative expression of *Fezf*, *Irx*, *Otx *and *Gbx *genes in the CNS is fully conserved between these species, suggesting a widespread involvement of these genes in early molecular patterning of the bilaterian CNS.

## Results and Discussion

### Fezf is highly conserved across phyla and is ancestral to bilaterians

**Figure 2:**
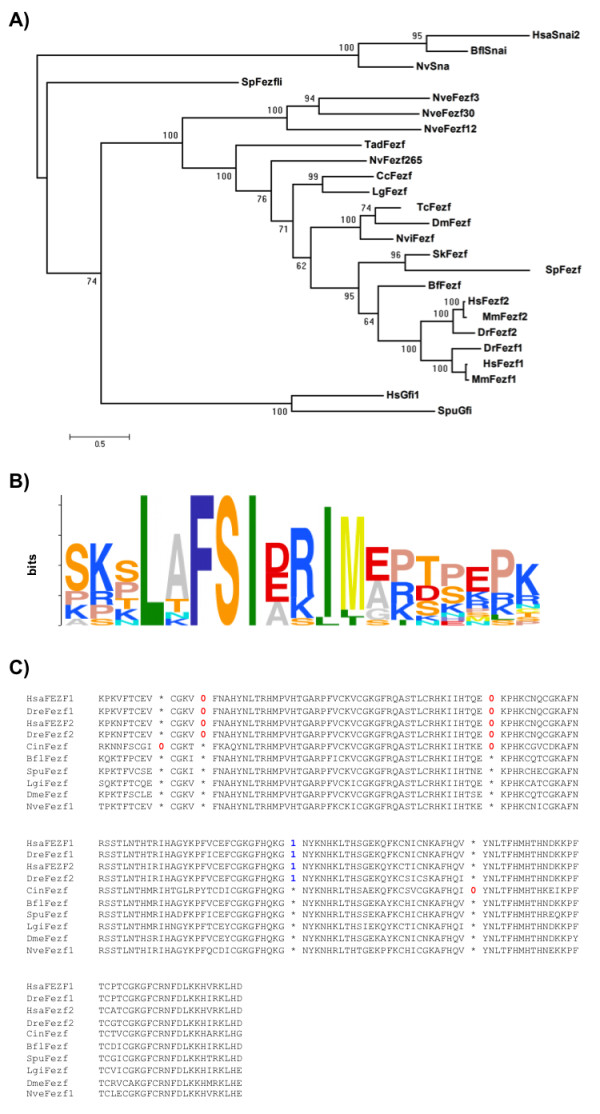
**Phylogenetic relationships, Fezf box, and exon-intron structure of Fezf genes across metazoans**. **(A) **Phylogenetic tree of the putative *Fezf *orthologs identified in different metazoan genomes generated by Bayesian inference. The orthology of the genes is supported by a posterior probability of 1. **(B) **Consensus sequence of the conserved Fezf box located near the C-terminal of all bilaterian *Fezf *orthologs. **(C) **Alignment of the zinc finger domains of some representative species showing intron positions and intron phases (colored numbers). Only vertebrates and *Ciona intestinalis *show lineage-specific introns in these domains.

*Fezf *is a transcription factor of the C2H2 zinc finger family, containing six zinc fingers. Using *in silico *analysis, we identified putative *Fezf *orthologs in all studied metazoans (see Methods), including non-bilaterians. The orthology of the different putative *Fezf *genes is robustly supported by phylogenetic analysis (Figure 2A). The coding sequences of the zinc finger domain are highly conserved between different groups, showing typically > 75% identity at the amino acid level. In addition to the zinc finger domain, non-bilaterians *Fezf *proteins have a co-repressor SNAG domain, typical of other related zinc finger gene families, such as *Snail *and *Gfi *[16]. This domain was probably present at the origin of *Fezf *gene family, but has been lost in all studied bilaterians, with the exception of amphioxus, for which we could identify a putative SNAG domain *in silico*, although reverse transcription PCR experiments showed that this domain is not included in the *Fezf *transcripts during development of either *Branchiostoma floridae *or *Branchiostoma lanceolatum*. Multiple convergent secondary losses of the SNAG domain have also been reported in the *Snail/Scratch *superfamily [16,17] and it has been proposed that these losses are associated with the acquisition of different conserved domains that carry out a co-repressor function, such as the CtBP-binding site or the NT box [16]. Consistent with this hypothesis, we identified a highly conserved sequence motif near the N-terminus of all studied bilaterian *Fezf *proteins, which we have termed 'Fezf box' (Figure 2B) and which seems to be exclusive to the *Fezf *gene family. The clear inverse relation between the presence of the Fezf box (in bilaterians) and the SNAG domain (in non-bilaterians and related genes) suggests that this previously unidentified conserved motif might also function as a co-repressor domain.

In addition to the canonical *Fezf *genes, we also found '*Fezf*-like' genes in cnidarians and sea urchin (*NveFezf3*, *NveFezf12 *and *NveFezf30 and SpuFezli *[18]); these genes branch at basal positions of the phylogenetic tree (Figure 2A) and contain a SNAG domain but not a Fezf box.

Finally, we also studied the exon-intron structure within the zinc finger domain, where the sequence can be confidently aligned. Surprisingly, whereas in nearly all species no introns are found within the zinc finger domain, all vertebrate genes have three introns (one conserved with the tunicate *Ciona*) that seem to be lineage-specific gains (Figure 2C). This is unexpected, considering the high conservation of intron positions from cnidarians to vertebrates in the deuterostome line [19-23] and the generally low rate of intron gains along these lineages.

### Expression of Fezf, Irx and Gbx in amphioxus

**Figure 3:**
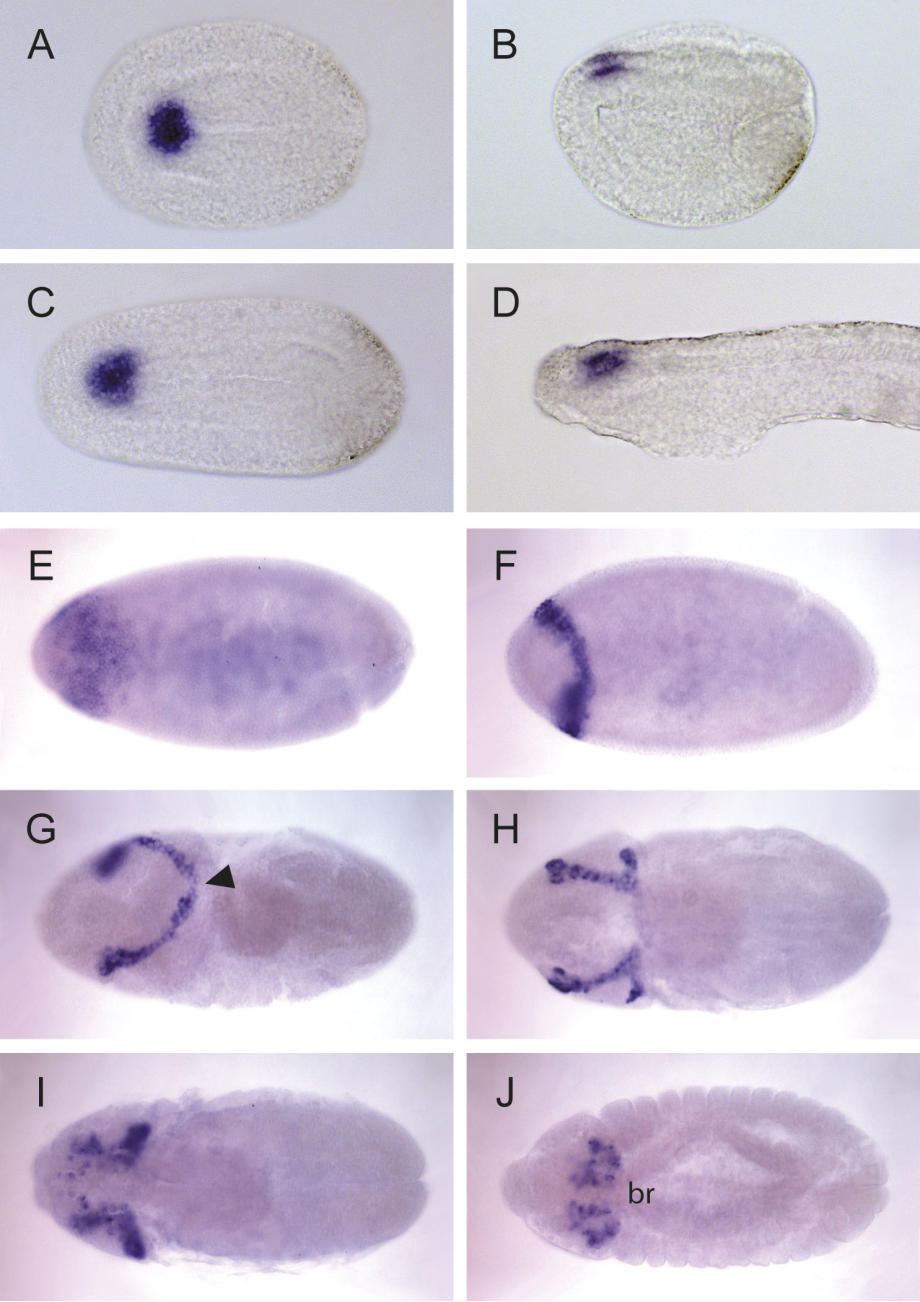
**Developmental expression of *Fezf *in amphioxus and *Drosophila***. **(A-D) **Expression of *Branchiostoma lanceolatum Fezf *(purple) during embryogenesis. **(A) **Dorsal view of an early neurula showing the highly restricted *Fezf *expression (purple) in the anterior neural plate. Expression is observed only in the anterior-most six to seven rows of cells in the neural plate. **(B) **Side view of the same stage shown in **(A)**. **(C) **Dorsal view of a mid-neurula stage, showing similarly strongly restricted anterior neural expression. **(D) **Lateral view of a premouth larva. Anterior is to the left and dorsal is up. **(E-J)**: Expression of *Drosophila Fezf *(purple) during embryogenesis, as previously reported [27], in embryos at stages 4 (syncitial blastoderm), 5 (cellular blastoderm), 8, 10, 11-12 and 14, respectively, as described previously [59]. Anterior is to the left. **(E, F, H-J) **dorsal views; **(G) **dorsolateral view. Arrowhead in **(G) **indicates the dorsal split of the initially continuous stripe. br brain hemispheres.

**Figure 4:**
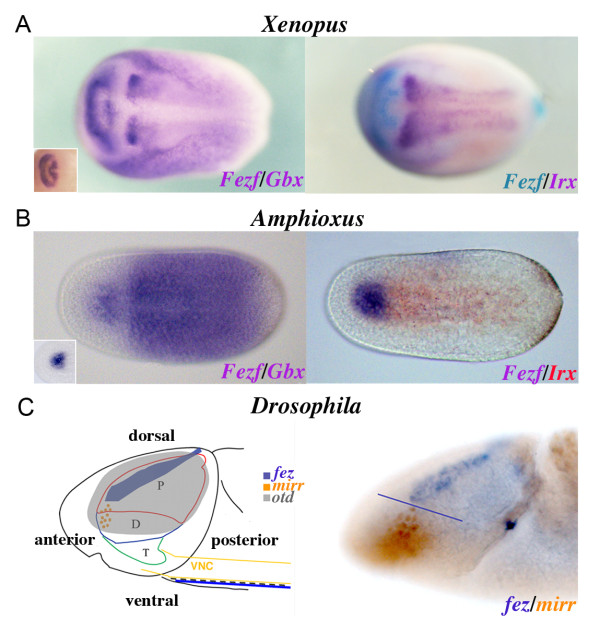
**Conserved basic CNS genoarchitecture in bilaterians**. **(A) **Expression of *Fezf *and *Gbx2 *in *Xenopus *neurula showing a gap between the expression of both genes (left) at the neural plate and expression of *Fezf *(blue) and *Irx1 *(purple) showing (right) abutting expression domains. **(B) **Expression of *Fezf *and *Gbx *in (left) the amphioxus neurula and expression of *Fezf *(purple) and *IrxB *(red) showing (right) abutting expression domains. **(C) **Schematic representation of *Drosophila *CNS (protocerebrum (P), deutocerebrum (D) and tritocerebrum (T) correspond in vertebrates with the forebrain, midbrain and hindbrain, respectively) showing the expression of (left) the *dFezf *(*earmuff*/CG31670), *Irx *(*mirr*) and *Otx *(*otd*) (*Gbx *(*unpg*) is expressed latter abutting *Otx *[12], and lateral view of the expression patterns of *Fezf *(purple) and *mirr *(orange). Anterior is to the left, dorsal views unless otherwise specified. Insets in **(A) ***Xenopus *and **(B) **amphioxus correspond to single *in situ *hybridization of *Fezf*.

To gain insights into the evolutionary origin of the vertebrate ZLI, we analyzed the developmental expression of the single *Fezf *gene of the basal chordate amphioxus (*B. lanceolatum*). As in vertebrates, *Fezf *expression starts at the beginning of neurulation, and its expression is highly restricted to the most anterior part of the neural plate (the six to seven anterior-most rows of cells) (Figure 3A, B). This restricted anterior neural domain continues to the larval stages (Figure 3C, D), at which point the expression is found only in the cerebral vesicle, the most anterior part of the amphioxus larval neural tube. We next compared *Fezf *expression to *Irx *and *Gbx *genes at neurula stage, which in vertebrates mark the posterior boundaries of the presumptive ZLI and MHB, respectively. Strikingly, we found that the relative expression of *Fezf*, *Irx *and *Gbx *genes in the neural plate is fully conserved between amphioxus and vertebrates (Figure 4A, B), indicating that these genetic interfaces, which contribute to delineate these major brain subdivisions, were present before the origin of vertebrates. In both *Xenopus *and amphioxus, the expression of *Fezf *abuts that of *Irx*, and there is a conserved gap between the expression domains of *Fezf *and *Gbx *that shows *Irx *expression (Figure 4A, B), consistent with an ancestrally conserved anterior-posterior topology of the MHB positioning relative to the ZLI. Moreover, the abutting expression of the *Fezf *and *Irx *genes constitutes a conserved genetic subdivision within the amphioxus presumptive diencephalon, raising the intriguing possibility of potential equivalents of proto-prethalamic and a proto-thalamic regions in the primitive chordate brain, consistent with other observations [24,25]. Further investigation will be required to assess to what extent these structures are homologous and functionally equivalent to their vertebrate counterparts or whether they correspond to distinct amphioxus novelties.

### Expression of Fezf in flies

To further investigate the evolutionary origin of these early genetic brain subdivisions, we examined the expression patterns of *Fezf *and *Irx *homologous in the *Drosophila *developing CNS. *dFezf/Earmuff/*CG31670 has been recently shown to maintain the restricted developmental potential of intermediate neural progenitors in *Drosophila *[26], and its embryonic expression pattern has been documented previously [27]. As in chordates, *dFezf *expression is restricted to the most anterior part of the fly CNS throughout early CNS development (Figure 3E-J). *Fezf *expression starts in blastoderm embryos as a dorsal and lateral stripe in the anterior (neurogenic procephalic) region of the blastoderm (Figure 3E,F). Characteristically, the lateral ends of this stripe widen, making the pattern resemble earmuffs (Figure 3G). In early germband extension-stage embryos, the stripe is split at the dorsal midline (Figure 3G), generating bilaterally symmetrical domains (Figure 3G-J). During later embryogenesis, *dFezf-*expressing cells delaminate and cluster to form part of the brain hemispheres (Figure 3H-J). Importantly, the expression domain of *mirror *(*mirr*), the earliest fly *Irx *expressed gene, also abuts that of *dFezf *(Figure 4C). Our results, along with the fact that in *Drosophila *the orthologs of *Otx *and *Gbx *also show complementary expression domains (Figure 4C, [12]), and the presence of the conserved gap between the expression domains of fly *Fezf *and *Gbx *orthologs, suggest that this simple initial genoarchitectural plan, which broadly subdivides the vertebrate nervous system, was present in the last common ancestor of extant bilateral animals.

Significantly, *Fezf *and *Irx *in vertebrates regulate each other in a mutually exclusive manner. In knockout or knockdown mutants for these genes in different vertebrate species, there is a shift in the expression limit of the counterpart gene, either anteriorly (in the case of *Irx *[13,14]) or posteriorly (in the case of *Fezf *[15]). To assess whether this situation was at least partially conserved in flies, we also analyzed the expression of *dFezf *in *iro^DFM3 ^*mutant embryos, which lack the *Irx *genes [28]. In stark contrast to vertebrates, we did not find any noticeable caudal shift in the posterior limit of *dFezf *expression (data not shown).

### Early complex brains

The strongly restricted expression of *Fezf *to the anterior forebrain in vertebrates is an indication of its crucial role in the patterning of the vertebrate brain. The presence of deeply conserved *Fezf *orthologs in all studied metazoans, from placozoans to vertebrates, thus raises the question of whether *Fezf *might play a similar conserved role throughout animal phylogeny, or whether it has been recruited for different developmental functions in the different phyla. We show that in two distantly related invertebrate groups with a centralized CNS *Fezf *orthologs are also expressed in a strongly restricted manner in the developing anterior CNS, suggesting that the ancestral function of *Fezf *in bilaterians might well be related to the patterning of the CNS. Furthermore, the conserved relative expression with other key patterning genes (*Irx*, *Gbx *and *Otx*) at early neurulation stages suggest that all these genes may help to define broad conserved regions within the neural ectoderm as a whole in different bilaterian organisms [11,12,29,30].

Based on several similarities in patterning gene expression and function, Reichert and collaborators suggested that the last common ancestor of extant bilaterians, Urbilateria, had a tripartite brain, and that *Drosophila *and vertebrate brains had a comparable anterior-posterior patterning [12,31,32]. These authors proposed a model with three domains consisting (from anterior to posterior) of: (1) forebrain/midbrain, (2) an intervening MHB region and (3) a hindbrain. These three structures are characterized by the specific expression of the *Otx*, *Pax2/5/8 *and *Hox *genes, respectively [12,31]. Our results complement and expand this model, adding an extra conserved genetic subdivision to the forebrain/protocerebrum of the studied species.

### Origin of the ZLI secondary organizer

In addition to *Fezf-Irx*, other sets of genes with mutually exclusive expression patterns have been proposed to be involved in the development of the ZLI in vertebrates. Based on misexpression analysis, it was first suggested that the mutual repression between *Irx *genes and *Six3 *(expressed at early stages in the whole anterior forebrain anlage, limited caudally by the anterior boundary of *Irx *genes) (Figure 1B[33]) contributed to the establishment of the ZLI and other diencephalic subdivisions [34]. In amphioxus early neurula, *BfSix3/6 *is expressed in the anterior-most part of the neural plate, with a posterior boundary seemingly consistent with that of *Fezf-Irx *[24]. However, the role of *Six3 *in establishing the ZLI has recently been challenged, because, in contrast to *Fezf, Six3 *expression is very dynamic and regresses rostrally, both in vertebrates and amphioxus [24,33,34], leaving a region free of *Irx *and *Six3*. *Six3 *function might thus be related to cell proliferation rather than to neural patterning at these stages [24].

Another important pair of genes with mutually exclusive expression patterns thought to be involved in ZLI formation in vertebrates are *Lfng *and *Wnt8b *[1]. *Lfng *is expressed widely in the chick prosencephalon, with the exception of a wedge-shaped area (presumptive ZLI), where *Wnt8b *is expressed (Figure 1B, [1]). This *Wnt8b*-positive/*Lfng*-negative region is where the ZLI will develop. In amphioxus, however, the single *BfFng *gene is expressed throughout the anterior-most part of the neural plate in early neurula, apparently with no discontinuity [35], whereas *BfWnt8 *is not expressed in the CNS at early developmental stages [36](Figure 1B).

Taken together, these results suggest that, although the genetic boundary determining the location of the ZLI in vertebrates was present in proximal chordate ancestors, some of the components of the putative ZLI gene network [1] were not yet assembled. Accordingly, molecules secreted as secondary organizers in vertebrates have not been found in the amphioxus developing brain [7,9]. Thus, it is likely that in vertebrate ancestors, the ZLI secondary organizers evolved through the recruitment of the expression of key morphogens to the cells located in the interface of these conserved major genetic domains, defined by the abutting expressions of *Fezf-Irx*. Presumably, this would have led to the development of new subdivisions and brain structures, possibly allowing the increase in proliferation and complexity of present-day vertebrate brains. However, it is also possible that internal brain organizers evolved before the vertebrates originated, and were then lost in the studied invertebrate chordates by reductive evolution. Equivalent signaling centers have not been reported in any invertebrate to date; however, it is still possible that a more thorough study of other invertebrates, such as hemichordates, which show a wide conservation of relative gene expression patterns with vertebrates [37], or basal slow-evolving protostomes, for which there are no molecular data yet available, will help to clarify the origins of the vertebrate brain complexity.

## Methods

### *In silico *Identification and comparison of *Fezf *genes across metazoans

Using the previously described vertebrate *Fezf *genes as queries, we performed tBLASTN and/or BLASTP searches against the genomes of *Branchiostoma floridae *JGI v1.0, *Trichoplax adhaerens *Grell-BS-1999 v1.0, *Nematostella vectensis *JGI v1.0, *Ciona intestinalis *JGI v2.0, *Daphnia pulex *JGI v1.0, *Lottia gigantea *JGI v1.0 and *Capitella teleta *JGI v1.0, using the JGI website (http://genome.jgi-psf.org/euk_home.html) *and *of *Strongylocentrotus purpuratus *Build 2.1, *Tribolium castaneum *Build 2.1, *Nasonia vitripennis *Build 1.1, *Drosophila melanogaster *Build Fb5.3, *Homo sapiens *Build GRCh37, *Mus musculus *Build 37.1, *Danio rerio *Build Zv8, using the NCBI website (http://www.ncbi.nlm.nih.gov/blast/Blast.cgi). For *Saccoglossus kowalevskii *we performed a tBLASTN search against the traces at NCBI and then manually assembled the genomic locus.

We then downloaded each corresponding genomic region and build different gene models using GenomeScan [38] and GeneWise2 [39] software as necessary. We compared these predictions with expressed sequence tags and existent gene models when available. Annotation and comparison of intron positions and phases across zinc finger domains was performed as previously described [40,41].

The amino acid sequences for the zinc finger domains were aligned using ClustalW [42] and the resulting alignment was manually curated. Phylogenetic trees were then generated by the Bayesian method, using the software MrBayes 3.1.2 [43,44], with the model Dayhoff+Gamma, recommended by ProtTest 1.4 [45-47], under the Akaike information and the Bayesian information criterions. Two independent runs were performed, each with four chains. For convention, convergence was reached when the value for the standard deviation of split frequencies stayed below 0.01. Burn-in was determined by plotting parameters across all runs for a given analysis: all trees before stationarity and convergence were discarded, and consensus trees were calculated for the remaining trees (from at least 1,000,000 generations).

Fezf box consensus was decided by the program Sequence Logo online (http://genome.tugraz.at/Logo/) using a multiple alignment for all studied species containing a Fezf box.

### Cloning of European amphioxus *Fezf, Irx *and *Gbx *genes

Primer pairs were designed to span the whole length coding sequences of the *B*. *floridae Fezf *[18] and *Gbx *[11] genes, if possible. A liquid cDNA library from different developmental stages of the European amphioxus (*Branchiostoma lanceolatum*) was screened by PCR using the *B. floridae Fezf *primers. *B. lanceolatum Fezf *and *Gbx *were cloned, sequenced and submitted to NCBI (accession numbers HM245959, HM245960; primer sequences: Fezf_L: ATGGCAATGTTCGGAACCCTTG, Fezf_R: TTACTCTGCGGCTGGAAGTG, Gbx_L: TGAAAATGCAGCGGCACAGC, Gbx_R: ATGCTGACTCCTCATGGCGAA). For *BlIrxB*, we used the previously reported full-length sequence [48]. Neural plate expression patterns for *Irx *and *Gbx *in *B. lanceolatum *were consistent with those reported in *B. floridae *[11,29]. To assess whether the putative SNAG domain was included in the transcripts we used the following primers: GCGACGGTTCCATAATTCGT (reverse, within the CDS) with M13F standard primer (for cDNA library amplifications) or ATGCCAAAGTCATTTCTGGTG (in the predicted SNAG domain) and. All bands were cloned and sequenced. A liquid cDNA library of *B. floridae *provided by G. Langeland and our own cDNA library of *B. lanceolatum *were used as templates.

### *In situ *hybridization in the different species, antibody staining and *Drosophila *strains

Antisense RNA probes were prepared from cDNAs using digoxigenin or fluorescein (Boehringer Mannheim GmBH, Mannheim, Germany) as labels. The *Drosophila Fezf *cDNA (GH 14092) corresponding to the CG31670 was obtained from the *Drosophila *Genome Resources.

*Xenopus *specimens were prepared, hybridized and stained as previously described [49,50]. For *in situ *hybridization of European amphioxus, we used a modified version of the protocol previously described [51] (see Additional file 1). Importantly, the hybridization temperature was 65°C, and antibodies were incubated for 3 to 4 hours, followed by overnight washes in MABT buffer (100 mM maleic acid, 150 mM NaCl, 0.1% Tween-20, pH 8) to reduce background. Detection was done with alkaline phosphatase-conjugated anti-digoxigenin (DIG) or anti-fluorescein antibodies. Alkaline phosphatase reaction products were visualized with nitroblue tetrazolium chloride (NBT)-5-bromo-4-chloro-3'-indolyphosphate *p*-toluidine salt (BCIP) (purple color), 2-(4-iodophenyl)-5-(4-nitro-phenyl)-3-phenyltetrazolium chloride (INT)-BCIP (red) or BCIP only (cyan). *Drosophila *embryos were collected on yeasted apple juice-agar plates [52]. Pretreatment of embryos and hybridization *in situ *were performed as previously described [53], with some modifications: proteinase K treatment was avoided and incubations with anti-DIG (1:1000) were performed for 1 hour at room temperature. For double *in situ *hybridization and inmunostaining, the rabbit anti-β-galactosidase (Cappel) antibody was incubated with the anti-DIG. First, β-galactosidase detection was carried out as described previously [54], then the *in situ *hybridization signal was developed as described above. The *Drosophila *strain used were *mirr^880-lacZ ^*and the *Irx *deficiency *iro^DFM3 ^*[55,56]. The deficiency *iro^DFM3 ^*was balanced over the 'blue' balancer TM6B, P{35UZ}DB1, Tb^1 ^(Flybase: http://flybase.org/). Embryos were simultaneously hybridized with probes against *dFezf *and anti β-*galactosidase *transcripts, and homozygous *iro^DFM3 ^*embryos were those not transcribing β-*galactosidase*. Embryos were dehydrated and mounted as previously described [57].

## Competing interests

The authors declare that they have no competing interests.

## Authors' contributions

MI conceived the study, carried out the expression experiments in amphioxus and participated in the sequence analyses. CP generated the *Drosophila *data. IM participated in the sequence analyses and performed the *Xenopus *and amphioxus ISH. JLGS conceived and participated in the design and coordination of the experiments and generated *Xenopus *data. FC coordinated the *Drosophila *experiments. JGF participated in the design and coordination of the project. MI, JLGS, FC and JGF wrote the draft manuscript, and all authors read, discussed and approved the manuscript.

## Supplementary Material

Additional file 1 ***Branchiostoma lanceolatum *ISH protocol**. Detailed protocol used for whole-mount *in situ *hybridization in the European amphioxus *B. lanceolatum *embryos Click here for file
