# Supplementary material for: Conserved developmental expression of Fezf in chordates and Drosophila and the origin of the Zona Limitans Intrathalamica (ZLI) brain organizer
Source: EvoDevo. 2010 Sep 1;1:7. doi: 10.1186/2041-9139-1-7 (PMC2942887; doi:10.1186/2041-9139-1-7)
Supplement: Additional file 1 — Branchiostoma lanceolatum ISH protocol. Detailed protocol used for whole-mount in situ hybridization in the European amphioxus B. lanceolatum embryos [file 2041-9139-1-7-S1.PDF]

B. lanceolatum ISH protocol

**Step**	**Minutes x times**
1- Rehydrate by washing to 40%EtOH-PBT	5'
2- Wash with PBT	5' x3
3- Proteinase K treatment: max. time (at 7.5ug/ml)	
- Gastrulae: 3'	
- Early-Mid Neurula: 5-6'	
- Late Neurula: 7.5-8'	
- Larva <36h 10-12'	
- Larva >36h 15-18'	
4a- Stop Proteinase K by adding 10ul of 10% Glycine	
4- Wash with Glycine 2mg/ml made in PBT	5' x2
5- Wash with PBT	5' x2
6- Re-fix with PFA in PBT	30'
7- Wash with 0.1M Triethanolamine[a] (pH 8)	5' x2
8- Incubate with 2.5ul/ml de Acetic anhydride in 0.1M Triethanolamine	5' (no shake)
9- Incubate with 5ul/ml de Acetic anhydride in 0.1M Triethanolamine	5' (no shake)
10- Wash with PBT	5' x2
(*Transfer to nests*)	
10- Wash with PBT	5'
11- Rinse with HybBuffer[b]	
12- Pre-incubate with HybBuffer	1-2h (>65ºC)
13a- Denaturize the probes	5' (70º)
13b- Hybridization	o/n (>65ºC)
--	
14- Wash with PREHEATED HybBuffer	10' (65ºC)
15- Wash with PREHEATED WashSolution[c]	15' x4 (65ºC)
16- Wash with MABT[d]	15' x2
17- Incubate in MABT + 2% blocking reagent (BR)	1-2h
18- Incubate in MABT + 2% BR + 10% Filtered Sheep Serum	1-2h
19- Incubate in MABT+ 2% BR + 1% Sheep Serum + Antibody	2-4h (or o/n 4ºC) 30rpm
19b- Wash with MABT	5'x3
19c- Wash with MABT	10'x5
--	
20- Wash with MABT	20'x5
(Transfer to 4-well plates)	
21a- Wash with MABT:AP Buffer (-Mg) (50:50)	5'
21b- Wash with AP Buffer (-Mg)	5'
21c- Wash with AP Buffer (+Mg)[e]	5'
22- Develop with filtered (0.80um) BMP or 2ul NTB + 3.5 BCIP per 10ml	
23- Stop and wash with MABT	5'x2
DOUBLE ISH:	
- Inhibit the alkaline phosphatase with MABT pH1, 10'	
- go to step 16	
24- Post-fix PFA	30' or o/n
25- Wash PBT	5'x3

REAGENTS

a- 0.1M Triethanolamine (pH 8.0) (10ml)
150ul de Triethanolamine
9.85ml H_2O
take to pH8.0 with HCl 1N (entre 100 y 200ul?)

b- HybBuffer (20ml)
10ml Formamide
5ml 20xSSC
400ul yeast RNA 50mg/ml
200ul heparin
400ul 50x Denhart
20ul Tween20
200ul EDTA 0.5M

c- WashBuffer (20ml)
10ml Formamide
5ml 20xSSC
20ul Tween20
200ul EDTA 0.5M

d- MABT 5x (1x needs 0.1% Tween20)
29.05g Maleic Ac.
21.94g NaCl
take to pH8.0 with NaOH

e- AP(+Mg)
1ml Tris pH9.5 1M
500ul $MgCl_2$ 1M
200ul NaCl 5M
10ul Tween20
